# Collectanea

**Published:** 1828-02

**Authors:** 


### COLLECTANEA.

Floriferis ut apes in saltibus omnia libant,

Omnia nos, itidem, depascimur aurea dicta.

### ANATOMY.

*Congenital Absence of the Gastrocnemii Muscles.*—A CHILD, seven years of age, complained of weakness and lameness of the left leg. From want of power in the limb to support her, she frequently fell. The bones of the leg were found perfect. The back part of the leg, where the calf is usually situated, was perfectly flat, arising evidently from the absence of the gastrocnemii muscles and their tendon. Beneath the skin were felt some tendinous fibres adhering to the bone, but considerably less than the tendon of Achilles. When an effort was made to extend the foot, and to rest upon the heel, the flexor muscles, not being opposed by the gastrocnemii, immediately reflexed the limb. This circumstance had previously been supposed to depend upon weakness or convulsive action of the leg, but the real nature of the case was now determined.—Two other similar instances are related by the same author. (PALETTA, *Exercitat. Pathologicæ*, vol. i.)

### PHYSIOLOGY.

*Case of Premature Growth.*—B. Eckhofer, born in 1806 of healthy parents: her mother had six children, all of whom had been very robust. At birth, she was twenty-three inches long, and weighed about two pounds more than usual. The fontanels of the cranium were not at all ossified. From the eighth day the mother's milk was found insufficient, and broth was given. A fortnight after birth, four teeth appeared; but, excepting the unusual size of the child, nothing extraordinary was remarked until the seventh month, when she was weaned, and, although her food was not of a very digestible quality, her health continued good. She now began to walk. The incisor teeth protruded through the gums, and the hair, which had hitherto been fair, became dark, and grew so fast as to reach the middle of the back. At nine months old she menstruated, and, in fact, exhibited every sign of puberty. The first time, the discharge continued for seven days, and occurred regularly until her death, which took place when she was twelve years of age. After the ninth month she did not for some time grow so rapidly, but at six years of age she was three feet nine inches in height, and weighed fifty-four pounds. It is remarkable that, notwithstanding the general development of the body, and of the organs of generation in particular, she never exhibited any sign of sexual instinct. (*Allgemeine Deutsche Leits. fur Gebactskunde.*)

*How to procure Animals of either Sex.*—M. GAROU DE BUZAREINGUES published, in 1825, some experiments relative to the reproduction of various domestic animals, more particularly of sheep. In a late Number of MAGENDIE's Journal, he has resumed this subject, and has related the result of some experiments made with two separate flocks of sheep. In addition to these, there are many observations on the same subject applied to mares and cows; but the most important relate to sheep.

A flock of sheep was divided into two equal portions, and a smaller or greater number of male or female lambs were to be produced, at the will of the proprietor, in each of these. The plan adopted in order to insure this result, was to employ very young rams in that division of the flock from which it was desired to obtain females; and strong and vigorous rams, of four or five years of age, in that from which males were to be procured. The first division was also recommended to have a more abundant supply of food, and more repose than usual, during the period of impregnation. The following table will show the effect of the first experiment:

| | *Sex of the Lambs.* | |
|---|---|---|
| *Age of the Mothers.* | Males. | Females. |
| 2 years | 14 | 26 |
| 3 years | 16 | 29 |
| 4 years | 5 | 21 |
| 5 years and upwards | 18 | 8 |
| Total | 55 | 84 |

At another farm:

| | Males. | Females. |
|---|---|---|
| 2 years | 7 | 3 |
| 3 years | 15 | 14 |
| 4 years | 33 | 14 |
| 5 years and upwards | 25 | 24 |
| Total | 80 | 55 |

Another experiment is thus related:—A flock of 106 sheep was divided into two sections of forty-two each, one containing the strongest ewes, of four and five years of age; the second, consisting of the weakest, either less than four or more than five years old: the first section was intended to produce a greater number of females than the second; and after having been marked, and placed in a good pasturage, four rams, of about ten months old, were turned into them. The other section received two strong rams, each aged more than three years. The remainder of the flock, making up the number of 106, belonged to the shepherds; they are generally stronger and better nourished than the rest, and these, forming a third section, were placed under circumstances similar to the second.

The result of the lambing was thus:

| | Males. | Females. |
|---|---|---|
| First section | 15 | 25 |
| Second section | 26 | 14 |
| Third section | 10 | 12 |
| There were four double births; two of which, in the first section, produced | | 4 |
| The two others, belonging to the second and third sections, produced | 3 | 1 |

It is to be remarked, that the lambs proceeding from she section in which the young rams were employed, were in all respects as fine as those begotten by the older and stronger rams.

In connexion with this part of the subject, we find, in another part of the communication, a remark of some importance. In 1825, twenty ewes, which had not borne for two years, received the rams clandestinely in the beginning of the winter; they were almost all of them remarkably fat; they produced sixteen females and four males. Among the number of these ewes were two old ones, which had been put up to fatten in 1824, but could not be sold because they were not in sufficiently good condition: these gave one male and one female.

M. Garou next carries his inquiries to the reproductive power in the mare and cow. Respecting the first of these he observes, that, wishing to obtain more female than male colts, he fed his brood mares on fresh food; that he chose for propagation only such as had not been foaled or even nourished by the mother the preceding year; and he did not give them the stallion until they gave evident signs of being in heat. Five mares, so chosen, produced five female colts; and, by following the same method, out of thirteen colts foaled that year, eleven were females; and one of the two males was the product of an old mare. He remarks, that some mares of a remarkably vigorous appetite always bring forth females; whilst those of delicate health as constantly produce males. The same remarks apply to the cow.

---

### PATHOLOGY.

*Talma's Heart.*—The great French tragedian died of stricture of the rectum, which reduced that intestine to the diameter of three lines, causing great accumulation above it, and ultimately rupture of the coats, with effusion into the peritoneal cavity. He laboured under another complaint, however, which must sooner or later have caused his death, and which is more interesting as having been more immediately connected with his profession. The heart adhered to the pericardium at its apex, forming a dark-coloured tumor, connected with the left ventricle, and which in reality was a true aneurism. The walls of this sac appeared to consist of the pericardium and serous membrane of the heart glued together, and containing layers of fibrin, while the muscular fibres of the ventricle, considerably attenuated, were partially extended over the tumor. The size of the aneurism was about that of a small egg; it had displaced the point of the left ventricle, which was thus thrown a little forward. The origin of this pathological condition of the heart, which was not suspected during his last illness, may perhaps fairly be dated from the following occurrence related by Talma's family:

Some years ago, after great exertion in playing the character of Hamlet, he suddenly felt a sensation of burning heat in the region of the heart, and an uneasiness which lasted one or two days, but which did not attract any serious attention. From this time he frequently suffered from palpitation, particularly after professional exertion.

It is probable that, in a mind like Talma's, passions, though artificially excited by the cunning of the scene, and the intense and absorbing manner in which he entered into the character he represented, may have produced the same effect upon his frame, as moral impressions from external causes are wont to do on others; thus assimilating in their effects the real and imaginary in the miseries of life. It is probable, too, that had this great tragedian not laboured under the obstruction which proved fatal, he would have expired in one of those tempests of passion which held the spectators in dreadful admiration, and thus (to borrow the enthusiastic language of his countryman) he would have died *au milieu de la gloire!* (*London Medical Gazette.*)

*Death from the Sting of Bees.*—M. Bertrand, thirty-four years of age, and of a good constitution, was attacked by a number of bees: they fixed upon his breast and face, causing considerable pain. He ran from the spot, but the bees pursued him, and continued to sting him. They were soon driven off, but he declared that he felt himself dying, and fell down almost immediately. He was found by the surgeon stretched upon a mattress, pale, and breathing feebly and interruptedly. His pulse was scarcely perceptible, and the surface of his body was cold. Under the extraordinary idea that this state arose from impending suffocation, and that a bee might have entered the trachea, the operation of tracheotomy was immediately performed; but the unfortunate gentleman died almost instantly, and not more than ten minutes after the receipt of the injury. No examination of the body was made.

---

*Artificial Anus.*—A woman, at the termination of a labour which happened twenty years before, had had an umbilical hernia, which, not being kept reduced, had gradually increased, till at length it acquired so considerable a size, that in the course of ten years the whole of the intestines appeared to be situated in a kind of pouch. At the end of the second year, the skin became red at the lower part of this, and ulceration took place, and in a short time a round opening was established, about the size of a five-franc piece. The circumference of this soon healed, but the centre of it remained pervious, and discharged pus, serum, and blood. During the ten years which had elapsed from the appearance of this ulceration to the time of her seeking medical aid, the aperture had healed up and opened again several times. The movement of the intestines could be seen through the integuments of the tumor. She was still able to continue her employment. Some time afterwards, however, she perceived that a portion of food which she had taken issued from the wound, three hours after it had been swallowed. Upon pressing the hernial sac, fæcal matter escaped from the opening. In the centre of the old wound there was an opening about the size of a shilling, the edges of which projected and formed a kind of sphincter. It was ascertained that this opening led into one of the small intestines. The intestine was reduced, and fixed near the wound, and perfect rest was recommended to facilitate the union of the parts. The stools passed in the natural way at first, but were soon suppressed, and the wound of the integuments contracted, so that an artificial anus was established. (*Archives Générales.*)

---

### PRACTICAL MEDICINE.

*Case of spontaneous Ecchymosis, with acute Subcutaneous Œdema.*—A female child, three years old, of a good constitution, and who had always enjoyed a perfect state of health, complained, immediately after taking a hearty meal, that she was incapable of standing. The legs were painful up to the knee. The left ankle was considerably swollen, and was the seat of numerous ecchymosis of a purplish colour. During the night, the right leg and foot presented the same appearances. The little patient was much agitated, and did not rest a moment. The skin was dry and hot, the belly tender. Next day, the upper eyelids became œdemaıous; the back of the left hand also swelled, and ecchymoses appeared in this part similar to those upon the legs.

The succeeding day, Dr. Ollivier visited her for the first time. The face was swollen, the upper eyelids œdematous, and so much tumefied as to prevent the globe of the eye from being visible. The left eyelid was not so much swollen as the right, but it was ecchymosed, and the purplish red colour of the skin did not disappear on pressure, as is the case in an erysipelatous blush of the surface. The back of the hands and the lower part of the forearm were œdematous, and covered with numerous ecchymoses of a deep colour, varying in size from a small speck to an inch and a half in extent. The inferior extremities had nearly the same appearance. The spots of ecchymosis were in these parts somewhat larger, and some were of a greenish yellow colour, as is observed when ecchymosis occurs from external violence. The child did not appear to suffer from pressure of the limbs, and the pain she experienced when the above symptoms first developed themselves had entirely ceased. The body was entirely free from ecchymosis. The pulse was full, and 120 in a minute. Mild aperients were given and laxative glysters were used, and the symptoms diminished in a few days. The tenderness of the belly still continued.

Again, however, the child suddenly complained of her feet being painful. They were very hot, much swollen, and very tender to the touch. Large patches of ecchymosis soon appeared about the ankles. Several glysters were given of decoction of poppy heads, and which relieved the pain in the abdomen. On the next day, the pain of the lower extremities was rather increased, and the spots of ecchymosis were more general. It was observed that the extravasation occurred almost instantaneously. The child was twice put into a warm bath, and the swelling of the legs and the appearance of ecchymosis were much diminished. The left hypochondrium was very tender to the touch.

A few days afterwards, the legs and thighs were covered with an eruption resembling the Urticaria Subcutanea of Willan, which remained but for a few hours, and was succeeded by fresh patches of ecchymosis. Four grains of calomel were given, which brought away some slimy evacuations with a little blood. The dose was repeated by the parents, with the same effect. The child had entirely lost its appetite, and, as it was now observed to have periodical attacks of fever, and the pain of the belly had ceased, four grains of the Sulphate of Quinine were given in a glyster. Fresh spots of ecchymosis appeared upon the extremities, and the child was restless and much agitated during the night. Upon consultation, it was determined to apply six leeches to the anus. The warm bath was repeated.

From this time the child gradually improved, although the œdematous swellings and ecchymoses of the skin still continued to appear occasionally. When it appeared to be in a state of convalescence, the symptoms twice returned with their original violence, without any apparent cause. By strict attention to diet, and the employment of laxative glysters, the little patient at length perfectly recovered.

At the commencement of this disease, there were no symptoms of intestinal irritation. The loss of appetite and constant pain in the belly, which soon came on, clearly show, however, that the digestive organs were deranged; and, from the simultaneous appearance of cutaneous symptoms with the abdominal irritation, it cannot be doubted that they arose from the intimate sympathy which is so well known to exist between the skin and the mucous membrane of the intestines. (*Archives Gén. de Médecine.*)

*Treatment of Traumatic Tetanus.*—The symptoms of this formidable disease in many cases do not appear until many days have elapsed after the infliction of the local injury. In such instances, Dr. WENDT recommends that calomel should be freely exhibited, so as to produce very abundant alvine evacuations. In this country purgatives have been often tried, but mercury has not been administered in the manner advised by Dr. W. In the first case he has adduced, the patient was attacked with tetanic symptoms fourteen days after a surgical operation. The jaw was completely locked; the body rigid. Two grains of calomel were given every two hours. The symptoms not being alleviated, six grains of jalap were added to each dose. The bowels were freely acted upon, and on the following day the extreme rigidity of the muscles was much diminished. The wound continued healing. The same doses of calomel were given, but at more distant periods. In four days the spasmodic symptoms had almost entirely subsided. A slight difficulty in opening the mouth alone remained, and this was relieved by friction. The salivary glands were not at all affected.

In another case, which also followed an operation, the patient was seized with tetanic symptoms after imprudently exposing herself to cold. Calomel was administered in the same manner, and in four days every alarming symptom had subsided.

Dr. Wendt has since seen many similar cases of trismus and tetanus, succeeding to local injury, and the alvine treatment, he states, has always been crowned with success. *(Journal Complementaire, &c.)*

---

*Sulphate of Quinine.*—M. BLAND, physician to the hospital of Beaucaire, has observed, in some cases of intermittents, that the use of this remedy, when given to the amount of twenty-four grains a day, produced considerable deafness on the second or third day of its employment. This effect was particularly observed in weak and nervous patients, who had laboured under tertian ague. The difficulty of hearing ceased on the eighth or twelfth day. During the deafness, the patients complained of rather severe headache. M. Bland suggests the propriety of being very cautious in the use of this valuable remedy in large doses.

---

*Fever.*—Dr. ALISON says, In all there have been 170 cases which I considered as Simple Fever, and among these only three deaths. All the three had delirium, and two of them had a certain degree of stupor: they differed only in degree from the next class of cases to be noticed, the degree of insensibility simply not being such as would have appeared dangerous, but for the sinking of the circulation, which was manifest long before death. In the only one of them in which dissection was permitted, no appearance was found that could be decidedly pronounced to be morbid. It may be observed, that under this head of simple fever a number of cases are included, in which there was apparently a threatening of dangerous complications at first, and which may probably have been reduced to the form of simple fever by the evacuations practised.

There have been in all seventy-nine cases in which the affections of the nervous system predominated, and evidently constituted a great part of the danger; and among these seventy-nine there were twenty-one deaths. But I have already stated, that even where the affections of the nervous system existed in a degree that seemed quite sufficient of itself to indicate danger, it very often happened that they were combined with such feebleness of pulse, and other marks of failure of the circulation, that death might be said to take place as much in the way of asthenia as of coma. In most of these cases the comatose tendency, with spasm and subsultus and the failure of the circulation, come on simultaneously and gradually; but in some there was a state of restless active delirium, with animation of the countenance, liveliness of the eyes, and contraction of the pupils, after the voice had become faint, the extremities cold, and the pulse very feeble, or almost imperceptible, constituting the condition which is described by Mr. Travers as "prostration with excitement," and which suggested the remark of Andral, that there is "dans plusieurs cas de fièvres graves, une excitation factice, aussi bien qu'une fausse prostration." This state generally subsided rapidly into absolute and speedily fatal insensibility. Of the twenty-one fatal cases, I considered only five to be examples of death strictly in the way of coma. The duration of the fatal cases under this head was from nine to twenty days, but seldom above fifteen.

We had dissections of fifteen of the twenty-one fatal cases under this head. The only appearance found in any of them which I could regard as morbid was effusion of serum, chiefly beneath the arachnoid and in the ventricles. In judging whether the quantity of serum found in these situations was to be accounted certainly morbid, I have taken as a guide the observation of Andral, that, in order to be so, the effusion should be "assez considerable *pour soulever notablement l'arachnoide*, ou *distendre les ventricules*;" but so far from finding, as he has done, such amount of effusion very rare, it has appeared to me the most frequent of all the morbid appearances observable in fever. In twelve at least of these fifteen cases, it seemed to me obvious that the arachnoid was partially elevated, and the ventricles distended beyond their usual dimensions. But the quantity of effusion was never great. What was found in the ventricles never exceeded half an ounce, and did not exceed two drachms in more than three cases. Farther, the quantity of this effusion bore no proportion to the degree of affection of the nervous system before death. In some who had these symptoms violently, it was well marked; but in a boy who died decidedly comatose, (his pulse being full almost to the moment of death,) it was very slight, not exceeding a drachm in the ventricles, and being hardly perceptible beneath the arachnoid. In a woman who had, among other nervous symptoms, repeated and general convulsions, it was equally inconsiderable; and in the case already mentioned of a young man who was jaundiced, and died quite comatose, with dilated pupil and stertorous breathing, the quantity found in the ventricles hardly exceeded half a drachm, and there was positively none beneath the arachnoid. Such an amount of effusion as is found in the heads of many fever patients may be regarded, I think, as a sign that an increased action of vessels has taken place there; but by no means as a proof that such increased vascular action has been the cause of all the nervous symptoms.

In the treatment of this form of fever, when seen from the commencement, I have no doubt that blood-letting is almost always a proper preliminary to all other measures; but after the pain of head has subsided, although the pulse be of good strength, its beneficial effects are less decided. The use of cold applications, especially to the head, and fomentations of the legs, and the pediluvium, are generally and justly esteemed. In some cases of active delirium, with pretty firm pulse, nauseating and purging doses of tartar emetic have appeared a very useful auxiliary. When the chief nervous symptoms are the comatose tendency and spasms, blistering the head and neck, and continued though not violent purging, appear to be the most suitable evacuations, and I have seen many striking instances of their efficacy. It may be worth while to state, in regard to purgatives, that they would appear to be useful chiefly on the simple principle of derivation, and the appearance of the stools can by no means be taken as a guide either to their use or discontinuance. I have many times used them for days together with what appeared to me manifest good effect on the nervous symptoms, although nothing in the slightest degree morbid could be detected in the evacuations; and, on the other hand, when the most urgent symptoms have been those of mere debility, I have withheld purgatives, although the stools were very dark-coloured and offensive, and have had the satisfaction of seeing the natural appearance restored, as the general febrile symptoms improved, without the use of any means specially directed to this end. The pulse, notwithstanding the fallacies so often attributed to it, has appeared to me a much surer indication of such strength of the circulating system as demands evacuation, than the heat of the skin. I have seen the heat, in various cases, as high as 103 and 104, when the pulse was soft and feeble, and the disease advanced, and when gentle stimulants appeared to me to be required, and were used with success.

When the nervous symptoms in the advanced stages of fever have been accompanied with manifest weakness of pulse, I have used wine in small but often gradually increasing doses, and camphor or ammonia, along with the evacuations now mentioned, or even, where the depression has been very great, without them ; and have thought them much more frequently and decidedly useful in the present epidemic than formerly. In reflecting on some of the fatal cases, I have thought that these remedies had been too soon given, and in others that they had been too long delayed; and I have no doubt, from watching the progress of many such cases, that errors may very easily be committed in both ways, even by persons conversant with the disease. The greatest difficulty has appeared to me to be in judging of the probable effects either of evacuations, or stimulants, or anodynes, in persons habituated to strong liquors, and whose symptoms in fever resemble those of delirium tremens.

Opiates have appeared of most essential service in some cases where there was much slighter delirium and restlessness, without pain of head or flushing, therefore generally after loss of blood. I have used them repeatedly in such circumstances so early as the fourth or fifth day, with this marked good effect, that sleep has followed their use; and, after one or two repetitions of the dose, the patient has passed into the state of drowsiness without stupor, which is known to be one of the most favorable forms of simple fever, and has had no farther occasion either for anodynes or other active remedies. The more violent tremors and subsultus of the advanced stage of fever have generally been combined with so much stupor as appeared to render the full use of opiates hazardous, and, where they have been tried under such circumstances, they have not appeared to me successful.

Of fifty-eight cases in which the function of respiration was embarrassed at one period or other of the disease, in a degree which appeared to indicate danger, thirteen were fatal; but of these, four only were fatal strictly in the way of asphyxia: in the other nine there was extreme debility, but no such urgent dyspnœa towards the close of life, as appeared from comparison with other cases, in which the pulse continued firmer, to be incompatible with the restoration of health. Indeed, in several of these last the breathing had become so easy many hours, or even days, before death, that the amount of disorganization of the lungs found after death excited much surprise. The duration of the fatal cases under this head was from twelve to twenty days. The cases of those who recovered from this complication were generally very protracted, in several instances lasting five, or even six, weeks before the fever subsided; after which, however, the recovery appeared to be complete.

Very few of the cases under this head showed any symptoms of pleurisy, and none who died during the continuance of the fever had the pleura inflamed. In two cases (a child three years old and a young man,) the affection of the breathing, which came on late in the disease, and when the strength was much exhausted, was decidedly croupy; and in both, on dissection, inflammatory thickening and tenacious muco-purulent effusion were found on the mucous membrane of the larynx, though not in such a degree as nearly to close the glottis. In general the affection of the chest appeared to be chiefly bronchitis, and this was sometimes evidently very general; but there was often great difficulty in forming a judgment whether or not the substance of the lungs was undergoing inflammation and disorganization, and how far evacuations ought to be carried in the hope of checking these processes; and this difficulty was by no means wholly removed even by examination after death. Dissection was obtained in two only of the four cases in which I have stated that death took place strictly in the way of asphyxia, and in neither of these was there any part of the lungs found so far condensed as not to crepitate when cut. In both the bronchi were much clogged with frothy mucus. In one this was the only appearance decidedly morbid. This was in a young man of slender make, admitted on the tenth day of the disease, and in a state of great debility: he was twice leeched on the breast, but not bled. In the other the lungs were very generally œdematous, and in several places even the superior portions were of a red colour. This was in a very strong young man, whose pulse was full throughout the disease, and who was largely and repeatedly bled both before and after admission, though not for a week before death.

In four of the cases which were considered as fever with affection of the chest, but in which the impeded state of the breathing did not appear to be the chief cause of death, we found, on dissection, pretty large portions of both lungs so far condensed as not to crepitate when cut, and smaller portions so heavy as to sink in water. In all these the condensation was chiefly in the posterior portions; and, consequently, the patients having all lain on their backs in a state of extreme debility for some days before death, it was difficult to say how much of the condensation was to be considered as the "peripneumonie des agonisans,"—that is, was to be ascribed to the congestion of blood in the depending parts of the lungs, during the time preceding death, when the strength of the heart may be supposed insufficient to counteract the effect of gravitation on the blood. But, although I have no doubt that part of the appearance was to be ascribed to this cause, and although a degree of the change alluded to may certainly be often seen in the posterior portions of the lungs when there has been no ground for suspicion of disease there, yet as the condensation was in each case somewhat greater in one lung than the other, and as it projected forwards towards the anterior surface of the lungs in some places more than in others, we concluded that the appearance might be regarded as indicating inflammation. I must add, however, that two of these patients (treated in Queensberry House) were seen almost from the beginning of their illness, and were bled repeatedly, and to as great an extent as the state of the general symptoms seemed to permit; and I am confident that no one, who saw the progress of the symptoms after the last bleeding in these two cases, would have thought us justified in the farther use of the remedy.

Besides early bleeding, leeching, and blistering, the remedies that have seemed to me most useful in cases of this complication have been small doses of tartar emetic, and of ipecacuanha and calomel, with opiates. Many of them have appeared to me to require an allowance of wine when they became protracted, and have improved under it.

The abdominal affection chiefly observed has been diarrhœa, with pain of abdomen; sometimes with tenderness; often with the florid red tongue; sometimes with bloody stools. Sometimes, however, pain of abdomen and vomiting have been the abdominal symptoms observed; and this was the case in the only one of these cases which proved fatal,—namely, in an old man, who had a very weak pulse and cool skin almost from the first. He had vomiting of matter like coffee-grounds, and died on the eighth day. The only decidedly morbid appearance found in him on dissection was enlargement and induration of the liver. The cases of diarrhœa with pain and tenderness were treated chiefly by small bleedings, general and local,—by fomentations and opiates, in such quantity as to moderate without abruptly checking the evacuations,—and by mild laxatives occasionally interposed when the stools were slimy and scanty. Some of these cases were very protracted.

In regard to the tendency to diarrhœa in fever, there appears to be a marked difference (perhaps not sufficiently known) between the inhabitants of France and of this country. It is stated by Andral, (Clin. Med. t. i. p. 12 and 14,) that there are few cases of fever in the hospitals of Paris in which a spontaneous diarrhœa does not occur, and that the greater number of these patients have from five to ten stools daily. I need not say that the opposite state of the bowels is most general in this country during fever, at least in adults. In children, painful diarrhœa is common; and the greater number of the cases under this head in the hospitals were below the age of fifteen. (*Edinburgh Med. and Surg. Journal.*)

---

### MEDICAL JURISPRUDENCE.

*On the Signs of Drowning.*—M. ORFILA lately read a Memoir on this subject to the Royal Academy of Medicine in Paris. The object is to establish certain criteria by which we may ascertain whether the submersion has taken place before or after death; and this he has attempted to do by numerous examinations of the bodies of individuals who have drowned themselves, and by experiments on dogs. The inferences which he draws are almost all of a negative kind, and, as they contain an epitome of all that is known on the subject, we subjoin them.

1. That the red, livid, and swelled condition of the face, with froth at the mouth and nostrils, which some authors have laid down as indicating that the submersion has taken place during life, leads to no such inference, as it is wanting in many who have been drowned, and is present in many who have met their death by other means.

2. That the same remarks apply to extreme paleness of the face, which is an effect of the body remaining long in the water. M. Orfila here describes the alterations which the skin undergoes in those who have been long submersed. He asserts that on the legs the integuments become indigo colour, and then brownish on exposure to the air, while the rest of the body is very white; but the moment it comes into contact with the air, it is successively converted into brown and green, commencing at the chest. Remaining long in the water also brings on abrasions of the skin, which may give rise to the idea of wounds having been inflicted.

3. Excoriations of the fingers and traces of dirt under the nails are of no assistance, because they are wanting in those who are drowned before they come to the bottom, while they may be present in a body which, being thrown into a river, strikes against various obstacles.

4. Injections of the brain and its membranes M. Orfila thinks would be a satisfactory indication of drowning, if it were proved that the body became cold in a vertical position. As, however, this sign is frequently absent in those who have been drowned, so it cannot be regarded as a positive proof.

5. In those who have been drowned, the right cavities of the heart, the venæ cavæ, the pulmonary veins and arteries, are generally distended by a quantity of black blood, while the left side of the heart and the aorta are much less filled; the right ventricle is of a blackish brown; the left of a clear rose colour; and the right cavities retain a less contractile power than the left. This condition, however, is met with in many cases of sudden death.

6. Although the blood is generally fluid, yet M. Orfila has seen it coagulated in one individual who was drowned; a fact also observed by Lafosse, and more recently by M. Avissard.

7. The dissection of more than fifty persons who had been drowned has satisfied M. Orfila that it is a mistake to suppose that in drowning the individuals die during inspiration, and that, in consequence, they have the diaphragm pushed into the abdomen, and the chest elevated.

8. He regards the colour of the abdominal viscera as indicative of asphyxia in general, but not from drowning in particular.

9. The experiments of Edwards, Jenner, Cox, and Orfila, show that water enters the stomachs of those who drown themselves; while this is not the case with regard to bodies thrown into the water. But, in order to give its full value to this sign, it would require to be proved that the water had neither been swallowed before death nor injected after it.

10. It is not true that the epiglottis is pushed down upon the larynx.

11. Great importance had been attributed to the presence of sanguineous froth in the windpipe. M. Orfila objects, however, that this exists in other cases, as in death from epilepsy and hanging; while it is wanting in the drowned who have remained long under water, without coming to the surface to breathe.

M. Orfila made a considerable number of experiments to ascertain if water enters into the ramifications of the bronchi of the drowned; and he has always found that the fluid entered the lungs of the animal, and that the quantity was greater or less in proportion to the care took to keep the head erect in lifting the animal out of the water. But the fluid is not found in the air-passages, if the examination be made soon after death. As to the question whether the water enters the lungs after death, the experiments of M. Orfila agree with those of M. Piorry, proving that it does enter, and more completely the more vertical the position of the animal; and consequently the presence of water is not a sufficient proof of drowning, as water enters the air-passages of the dead body, and that the only absolute sign is the presence of water in the minute ramifications of the bronchi; it being proved that this water is the same as that in which the individual was immersed, that it had been injected after death, and could not have penetrated to the lungs from the vertical position of the body in the liquid.

---

### SURGERY.

### EXTRAORDINARY OPERATIONS.

*Aneurism: Application of a Ligature beyond the Tumor.*—In a recent Number of the American Medical Recorder, we find the following passage:—"We may be permitted to state our belief that persons have been committed to the grave because improper surgical measures (surgical operations) were enforced, in cases where ignorance alone was not a sufficient plea, but where daring arrogance, unfeeling boldness, and a longing after fame, have hurried on to the commission of acts which would sully the bright escutcheon of any one guilty of violating that sacred charge—'Thou shalt not kill.'"

From this extract it would appear that our transatlantic brethren are not behind in the practice of what, among us, has received the gentler appellation of "bold surgery; and, although we would neither encourage timidity, on the one hand, nor, on the other, apply such strong language to any thing that has been done among us, yet we look upon a passion for performing extraordinory feats in surgery as one of the characteristics of the present day. We, therefore, purpose examining some of those operations which have of late been proposed, with a view of fairly ascertaining the grounds on which they lay claim to our confidence; and we are the more impressed with the necessity of making this scrutiny, from observing that the usual mode of reasoning on such subjects has, in some recent discussions, been lost sight of, or altogether abandoned. Formerly, the cure of the disease, and the recovery of the patient, were looked upon as somewhat in favor of the treatment adopted; and, on the contrary, a fatal issue was thought rather to militate against the efficiency of the means employed. Indeed, it was reserved for the last few years to bring to light examples at variance with these old-fashioned notions; and it would now appear so little requisite that there should be any connexion between the remedy and the complaint, that operations are represented as having cured diseases which dissection has proved never to have existed: while so little has recovery to do with success, that in one instance the cure was published when the patient was in her coffin. had perfectly cured.

Events of this nature are calculated to mislead the profession, to retard the progress of science, and to bring discredit on our art: we shall therefore, in the present and some future Number, discuss the subject on the principle stated in our Address,—that, when men likely to have influence were "disseminating opinions or acting on principles practically injurious, we should do all in our power to obviate such an evil."

The first of these operations to which we shall advert, and probably the only one which our limits will permit us to discuss in the present Number, is that of tying the artery beyond the tumor in certain cases of aneurism. This method has been revived by Mr. Wardrop, after it had failed in the hands of one of the most eminent surgeons in Europe, (Sir Astley Cooper;) a circumstance which ought to have led to considerable caution in any attempt to bring it forward again. We think this remark will be justified by the examination, which we subjoin, of those cases in which it has recently been performed by Mr. Wardrop.

The case in which Mr. Wardrop originally performed the proposed operation, is detailed by him in the 13th volume, part 1, of the Medico-Chirurgical Transactions, and in the following words:—

"A lady, seventy-five years of age, after a very violent fit of coughing, perceived a swelling on the right side of her neck, a little above the clavicle. When I saw her eight days afterwards, the tumor had all the characters of an aneurism of the carotid artery, and had become as large as a fist, but was so situated that it was quite impracticable to tie the vessel below the tumor, so closely did it come in contact with the clavicle. The tumor continued to increase in size; and, *on the eleventh day after it was first observed, it had acquired a formidable aspect, the scapular portion having become very red and painful,* the pulsation, which was very strong throughout the whole swelling, being here particularly so, and the parietes *feeling extremely thin, and as if ready to burst.*" After the operation, the size of the tumor, and the strength of the pulsations, gradually diminished. "The redness of the skin, however, continued to increase; and that of the scapular portion of the tumor to become more and more of a purple colour, till at last ulceration commenced on the most prominent part. Several considerable sized portions of coagulated blood were discharged, *along with some healthy pus,* through the ulcerated opening; and, on the twentieth day after the operation, the ulceration of the integuments had closed, and nothing of the tumor remained but some wrinkling of the skin, and a considerable degree of thickening of those parts on which the base of the tumor had rested."

Now, we ask, who ever heard of an aneurism which, at the end of eleven days from its commencement, had advanced so far as to be inflamed and ready to burst? Who ever heard of an aneurism which, within three weeks more, did actually ulcerate and discharge healthy pus, mixed with some coagula of blood? It is impossible, we think, for any practical surgeon to read this case, and to look at the drawing given of it by Mr. Wardrop, without perceiving that the whole history is at variance with the obscure origin,—the gradual development, and protracted course of aneurism. We believe that the nature of the disease in this instance was altogether mistaken. And, at all events, giving to the case the most favorable construction of which it is susceptible, we hesitate not to say that it is so different from other cases of aneurism, as to lead to no rational inference with regard to the effect of similar treatment in that disease.

The next case is that of Mrs. D., who laboured under aneurism of the arteria innominata, for which Mr. Wardrop tied the subclavian artery: and the point to be ascertained here is, not whether there was really an aneurism, as in the preceding case, nor whether the vessel was bonâ fidê tied, as in that which is to come: but the question is simply this,—what is the present condition of the patient?

Dr. Barry, in some observations which he lately made in public, adverted to this case, stating that he had seen and minutely examined the subject of this operation, and that the result of the examination had satisfied him that she laboured under organic disease within the chest, which gave rise to a distressing difficulty of respiration, and placed her in a very precarious condition. This statement was made, not only without any expression of disapprobation towards Mr. Wardrop, but in the most fair and handsome manner. There is something, however, intoxicating in flattery, and those who long have been "dieted on praises sauced with lies" cannot relish wholesome food. Mr. Wardrop was induced to write to the editor of the "invaluable" Journal a letter, which places on record his account of the state of Mrs. D. at a particular period, to which account he thus stands pledged. He informs us, under date December 5th, that Dr. Barry had given "a very imperfect statement," and that he was anxious to prevent the public from being misled by it. He then proceeds to inform us, that his patient was in the enjoyment of "good, though feeble health," and that "a perfect cure had been effected." Now, in judging between the opposing statements of these gentlemen, it is of importance to be aware that Dr. Barry had been frequently with the patient, and founded his opinion upon actual examination. Whereas Mr. Wardrop, at the time he wrote his account, had, as we are informed upon unquestionable authority, not seen Mrs. D. for several weeks. And we assert that, so far from her enjoying "good, though perfect health," and *à fortiori*, so far from a "perfect cure having been effected," she is at this moment labouring under thoracic disease, which, if not accelerated, has at least been in no degree retarded by the operation, and that she is now in apparently desperate circumstances.*

Dr. Barry, in a letter, the delicacy of which towards Mr. Wardrop forms a striking contrast to the treatment he had himself experienced, well remarks, that "it is of the last importance to the profession, and to humanity, that the principles and the application of this bold and novel practice should be maturely and carefully weighed. That this hazardous operation should be viewed in all its bearings by other eyes than those of the man whose zeal for the improvement of chirurgic medicine first led him to perform it, but whose cooler judgment may for a moment have been dazzled by the brilliancy of the anticipated results."

Of Mr. Wardrop's remaining case, little need be said. It was supposed to be an aneurism of the carotid artery, and Mr. Wardrop was supposed to have tied that vessel; but, as both these suppositions proved to be erroneous, the operation, in whatever it may have consisted, can have no influence on the present question.

There is yet one other case to which we have to allude: it is that which fell under the care of Mr. Lambert. This was a very small dilatation of the carotid near its origin; and we are informed by the author himself "that Sir Astley Cooper discountenanced the operation, and remarked that it was an aneurism by dilatation, which would not increase." Mr. Key, Mr. B. Cooper, and Mr. Callaway, severally gave opinions against the operation. Mr. Lambert, however, acknowledges that he "was not at all satisfied," and consulted his "friend Mr. Wakley," who "recommended the operation to be immediately performed;" and, lastly, Mr. Wardrop "unreservedly declared himself" of the same opinion. Mr. Lambert, backed by these authorities, proceeded to the operation; and thus afforded the first practical illustration of the evil which has resulted from the view given of these cases, and the premature conclusions which have been drawn from them. The patient died, in consequence of the artery ulcerating at the point where the ligature had been applied. This case, however, unfortunate as was the result, and imper-

* The aneurism is now increasing externally, so that the patient is much in the same state as before the operation.

fect as is the evidence it affords, yet goes farther to establish the principle than any of Mr. Wardrop's; because dissection showed that a restorative process had begun, and thin layers of lymph were discerned on the inner surface of the dilated part of the vessel.

Mr. Wardrop, in the letter alluded to, says he has reason to believe "that not only the principle is now universally admitted, but that in future the operation will, without hesitation, be adopted." With all deference to Mr. Wardrop, we think that, after the examination of his cases which we have just made, surgeons will hesitate very considerably; and, before they either admit the principle or follow the practice, they will require some evidence less equivocal than that of four operations, in one of which the carotid artery appears to have been tied indeed, but where we hold that there was no aneurism; in the second of which, dissection showed that there was neither aneurism, nor had been ligature of the vessel; in the third of which, we have speedy death; and in the fourth of which, the physician in attendance (a friend of the operator) declares that he has "strong motives for reserve" as to the "perfect propriety and complete success of this operation." Had the account of this last rested on our authority alone, it might, and no doubt would, have been met by denial; but as it is, Mr. Wardrop can only exclaim, in the words of the Spanish proverb, "God defend me from my friends, and I will defend myself from my enemies." Assuredly no one had ever more cause to make use of the exclamation.

[The operation has likewise been performed by Sir E. Home; who, induced by the alleged success of Mr. Wardrop's first case, tied the inguinal artery in an aneurism of the external iliac, at Chelsea College, but without any effect whatever in arresting the progress of the disease.]

*Extirpation of Ovarian Tumors.*—There is a curious contrast between the medical and surgical practice of our continental brethren,—the former employing such remedies as marsh-mallow ptisans and orange-flower water, and exclaiming against the practice of England as fit only for horses: the latter ventures on operations so daring, that most English surgeons shrink from performing them. Of late years, however, some of the operators of this island have shown an anxiety to import such operations from the continent, or to invent others which vie with them in boldness. Mr. Lizars, of Edinburgh, published four cases, in which long incisions had been made into the abdomen, from which the bowels protruded, for the removal of tumors of the ovary. Of the four patients who were the subjects of this operation, one died, (we do not like to use a stronger expression;) in another, when the abdomen was laid open, no tumor was found; in the third, the tumor adhered so extensively that it could not be removed; in the fourth, one tumor was removed, but another, almost as large, was left behind. One would suppose that these results would have been sufficiently discouraging, yet Mr. Lizars takes for his motto this sentence: "If English surgeons will not perform these operations, those of France and America will." Although Mr. Lizars has formed an estimate of these cases exactly the opposite to that of ninety-nine men out of a hundred, he deserves great credit for the candour of his publication.

We understand, on authority which we cannot doubt, that, within the last month or two, a practitioner of note in this town has extirpated the uterus of a poor woman from the country. What the result was, we know only from report, but we think, in honesty to his brethren, he is bound to publish it.

Whether successful or not, it will be a useful guide to those who are deliberating about the propriety of performing this operation. Nothing can be more mischievous than to publish only fortunate cases, except it be to permit failures to circulate as successes. A few months ago a paragraph appeared in the Literary Gazette, headed "Extraordinary Surgical Operation," stating that a tumor, eight pounds in weight, and larger than a man's head, had been extracted through an incision nine inches long, from the abdomen of a woman, with great facility, and with the loss of not more than two ounces of blood. Since then it has been copied into the New Monthly Magazine. What will our readers think when we tell them that the poor woman died a few days after the operation; yet this important appendix to the case has never been added to it, and it has circulated as a successful operation among the numerous readers of the Literary Gazette and the Monthly Magazine; many of them, of course, medical men, and some of them young surgeons, longing to flesh their knives with some such surgical enterprise.

*Method of bringing on Premature Labour, and preventing future Pregnancy.*—The next operation to which we would call the attention of our profession, is one which Dr. Blundell, the lecturer on midwifery at Guy's Hospital, is represented as recommending to his class. It is——but our readers will not believe it unless we give his own words, which we shall do as they are reported in the Lancet. Speaking of those cases in which the pelvis is so narrow that at the full time it is impossible to extract the child, excepting by the Cæsarian operation, the Doctor says—"Suppose a woman has a high contraction, (an excessively contracted pelvis?) and is in the early months of gestation; I will suppose that she may not have gone above one or two months: now in this case, of course, it would, if practicable, be desirable to introduce an instrument into the uterine cavity, so as to discharge the liquor amnii, and in that way bring on premature delivery; but very probably you might not be able to enter the uterus: *nay, you might not be dextrous* or fortunate *enough even to feel the os uteri.* Under these circumstances, another operation, and an operation which *I would strongly recommend* to your attention, might be attempted. Make an opening a little above the symphysis pubis, in or near the linea alba, carefully avoiding the bladder: at this opening introduce one of your fingers, say the forefinger of your left hand, so as to get a bearing (?) on the uterus. This accomplished, take some slender-pointed instrument and pretty stiff, and, by a sort of acupuncture, carry this instrument through the body of the uterus into its cavity, and, on entering the uterine cavity, move the wire cautiously, yet effectually, in different directions; so as to break the ovum all to pieces, and put an effectual stop to the generative process. The ovum destroyed, draw up the fallopian tube, which is easily done, first on the one side, and then on the other, cutting out a portion of it so as to render it impervious, by which the woman would for ever afterwards become sterile. By this operation, successfully performed, you would at once secure the woman against the Cæsarian incision, and preclude the risk of her ever being pregnant again. In performing the operation, I should be very careful to break up the ovum thoroughly, even if I laboured fifteen or twenty minutes."

Thus, unless the reporter has made some mistake in his account of the lecture, which we sometimes suspect, an operation is recommended to an ordinary practitioner of midwifery, which few hospital surgeons would venture to perform,—namely, to make an incision into the abdomen; to thrust a pointed instrument through the uterus into its cavity; to move it about, and thereby destroy the texture and life of the ovum; to draw out the fallopian tube and cut off a portion of it, and then serve the other in the same way;—and this operation is put into the hands of a man who is supposed to be such a bungler as not to find the natural orifice of the uterus! Making full allowance for the difficulty of feeling the os uteri in the first month or two of pregnancy, and supposing this difficulty to be increased by the excessive deformity of the pelvis, surely it would be better to take a lesson from our continental brethren, and dilate the vagina by a tin speculum, and light it up by a candle to help us in our search. Any plan to avoid such—let us use the doctor's own words—such "a ferocious, atrocious" operation.

We close this article with the account of an operation, of which the hardest thing that can be said is, that it is rather whimsical. Dr. Arnott, in his late work, entitled the Elements of Physics, proposes as a substitute for the midwifery forceps an instrument like a schoolboy's sucker, which he denominates his Pneumatic Tractor. It consists of a circular piece of leather, three inches in diameter, and kept extended by a ring, with a cord fixed firmly to the centre: this is to be wetted, and applied to the presenting part of the child's head, just as the schoolboy would apply his sucker to a flat stone; by extending the cord, and thereby raising the centre of the leather from the head in the one case, or the stone in the other, a vacuum is formed between the leather and the head, or stone, and the surrounding edge grasps it firmly like that of a cupping glass. "This (says Dr. Arnott,) would act upon any body, to lift or draw it, with a force of about a hundred pounds, and with much more, therefore, than is ever required or allowable in obstetric practice."

The most ungentle criticism which we have to direct against the proposal of this agreeable writer, is a smile: but how easy would it be to put this proposal to the test of experiment, when perhaps the laugh would be turned against ourselves. We heartily wish it might; for certainly the midwifery forceps are safe and useful only in the hands of those few who employ them often enough to acquire dexterity in their application. Be it remembered that the forceps have two powers of essential importance in the cases for which they are used: the one that of slightly compressing the head, the other that of turning it from an unfavorable position. Neither of these are possessed by the Doctor's tractor, which we think much more likely to tear off the scalp than to pull out the head. (*Medical Gazette.*)

---

*Spina Bifida cured by Puncture.*—A child was taken to Professor Ruggieri, with paralysis of the inferior extremities. Upon examination, it was found that spina bifida existed upon the lumbar vertebræ. The tumor was transparent, and painful upon pressure. There was an obvious fluctuation in it. The mother was desired to protect it from injury as much as possible, and no curative means were attempted. The child was shortly afterwards entrusted, perfectly naked, to the care of its sister for a few moments, and by some accident the tumor was punctured. A large quantity of fluid escaped, and the child suffered no inconvenience. It was taken almost immediately to M. Bozeki, who found the tumor flaccid and wrinkled. Slight pressure was applied by means of a bandage; but in a few days the tumor again enlarged, although it did not regain its original size. It was now punctured with a needle, and its contents evacuated. Slight inflammation arose on the following day in the tumor, and adhesion took place between the skin and surrounding parts. A small quantity of fluid again collected, which was removed in a similar manner. Compression was again applied, and the surrounding parts became completely consolidated. In the course of a year, the vertebral column had acquired a semi-cartilaginous hardness, and the paralytic affection of the inferior extremities was entirely removed. A thin sheet of lead was kept upon the part by means of a bandage.

Another child was treated in the same manner, and with similar success.

The relator of these cases observes, that so fortunate a result must not be expected when spina bifida is complicated with hydrocephalus. *(Annali Universali di Med.)*

---

*Treatment of Nævi by Ligature.*—Mr. Lawrence, after enumerating several cases successfully treated by ligature, makes the following remarks :—

The superior safety of the ligature, in reference to the danger of hemorrhage, is rendered obvious by these cases. In the first I would not have performed excision, feeling satisfied that immediate death from bleeding would have been inevitable. There would have been less risk in leaving the case to nature. But, supposing this danger to have been surmounted, the patient would have been in a much more dangerous state with the great wound of excision, than with the comparatively small breach of surface caused by the ligature. It would have been necessary to make an elliptical incision, with each side at least four inches long. The ligature is applied close to the base of the tumor, while it would be necessary, in order to clear the diseased part, to keep half an inch from it with the knife. This is an important advantage on the side of the ligature in the numerous instances where extensive loss of skin would cause deformity.

The knife might have been safely used in the second case ; but the operation would have been serious, and the loss of blood considerable ; while with the ligature the latter was entirely avoided, and no symptom of the smallest consequence occurred.

The treatment by excision in the third case would have involved the necessity of a very large wound, and the risk of fatal hemorrhage. The latter danger would have equally attended the same practice in the fourth instance : a large wound would have been required, running very near to the edge of the lower eyelid, with a great probability of eversion on the contraction of the cicatrix.

Our object, in this mode of treatment, is to intercept the circulation completely in the tied part, and thus to deprive it of vitality as speedily as possible. The ligatures must be drawn very tightly, and ought therefore to be so strong as to bear the utmost force we can employ with the fingers and thumbs without breaking. Stout silk twist will answer the purpose ; but we should observe the precaution of trying its strength previously. As it is necessary, in some instances, to include a large bulk of parts, their complete strangulation is not easily effected, however strong the ligature may be. Perhaps it might be advisable, under such circumstances, to adopt the proceeding recommended by Mr. Mayor, of Lausanne.

As the continued pressure of the ligatures keeps up irritation in the parts immediately constricted, and in those around, which are forcibly dragged together and puckered, it is desirable to remove them as soon as we can be certain that the objects of their application are accomplished,—namely, coagulation of the blood in the vessels composing the tumor, and death of the part. The foregoing cases prove that these purposes are fully effected in forty-eight hours, while the fourth shows that we cannot safely trust to a compression of one half that duration. The danger of inflammation or irritation is at an end as soon as the ligature is taken away, and the relief to the patient from its removal is very obvious.

Mr. ANTHONY WHITE, on the same subject, says,—

It has been my practice during many years to destroy the larger specimens of nævi by strangulation with the ligature: a curved needle is passed under their base, and a single or double ligature used, according to the magnitude, figure, and situation of the tumor; and I recollect one example, in the destruction of which it was necessary to pass four ligatures in order to entangle the whole of the spurious structure, on account of its irregular form and diffused surface, situate behind the ear and extending down the neck of a very young infant. An assistant should raise the tumor with as much of surrounding skin in which it grows as possible, and thus enable the operator to pass the needle sufficiently deep, and at the same time include with his knot a portion of the healthy integument.

When the nævus is not large, a needle of sufficient length being carried under the centre of its base and the structure elevated upon it, a single ligature passed behind the needle, and firmly tied with a double noose, will be sufficient to destroy the organization. Where, however, the size of the tumor renders its strangulation by this mode doubtful, the needle should be armed with a double ligature, to enable the operator to tie one half with each. This, in every instance where the whole substance has been included, has uniformly destroyed the structure, leaving, on its separation, a healthy granulating surface. There are some instances, however, and some situations, where it is impossible to include the whole of the tumor with the ligature; but generally, in such cases, the inflammation excited by its application in the neighbouring parts is sufficient to obliterate, by condensation of the surrounding cellular structure, any remaining portion of the nævus.

I have met with but one instance of a partial recurrence of the disease, but which I had anticipated. The history of the case is detailed hereafter. (*Medico-Chirurgical Transactions.*)

---

*Fungous Eruption.*—Mr. WALLACE gives the following account of a disease of this nature curable by mercury, but not of venereal origin.

Martin Lavin, aged forty years, of a tall and slender figure, with a pallid countenance, a poor man, earning a livelihood by a traffic in old clothes, broken glass, &c. applied at the Skin Infirmary, on the 16th of October, 1819, to obtain relief for a disease, of which the following is a description.

A number of tumors, some about the diameter and elevation of a filbert, others as large as a small walnut. On an accurate examination, they were observed to be crusted, or covered with a yellow brown scab, and to be surrounded, to the extent of about two lines, by an areola of livid-coloured skin, which was rough, in consequence of desquamation of its cuticle. On removing the scab or crust, it was found to form a covering or cap to a fungus or excrescence, of a pale or dirty pink colour, having but little sensibility when handled, and exactly resembling in figure a small mulberry or raspberry. There were also, on many parts of the surface of the skin, marks or stains of a rounded form and livid colour, of the same diameter in general as the fungi, scarcely elevated above the surrounding skin, wrinkled, and in some situations with the cuticle covering their surface in a state of furfuration.

Each of the existing fungi, and several others which he says have been cured, commenced (according to his description) in an itchy pimple, which gradually increased without much soreness or itching; and having existed for some time, many imperceptibly shrunk away, leaving behind them the livid marks above mentioned. Nevertheless, it does not appear that they spontaneously subsided, but in consequence of unguents which he applied to them.

The fungi at present in existence are five in number, and are seated one in each of the following situations,—viz. in the nape of the neck, close to the hairy scalp; on the left of the anterior part of the abdomen, a little above the umbilicus; on the anterior fold of the right axilla; on the corresponding arm, immediately above the internal condyle of the humerus; and on the upper and outer part of the right thigh.

There is no pain in any of his joints or bones,—no soreness of his throat,—no perceptible derangement of his general health.

He attributes the origin of these fungi, which he says commenced in last April, (six months previously to his application at the Skin Infirmary,) to lying in dirty beds. Has a wife, with whom he cohabits, and has not communicated the disease to her. He never saw any person labour under a similar disease. Denies having ever had gonorrhœa or chancre. Has not had his health deranged in any way, either immediately before the appearance of the fungi, or at any time since.

One of the most remarkable circumstances connected with the growth of these fungi, is the power which the part upon which they are seated possesses of healing, without the formation of any permanent cicatrix: demonstrating that these fungi grow from the *surface* of the cutis, and that the texture of this covering is not permanently injured. Indeed, if the fungus be healed before the process of ulceration has commenced, no mark whatever, except a temporary redness, with a disposition to furfurate, remains; but, if ulceration has taken place, a permanent cicatrix is necessarily produced. I have, however, on many occasions remarked a fungus to heal without either ulceration or a permanent cicatrix, after it had arrived at the magnitude of a walnut. While observing the progress of cicatrisation, I have remarked that, although it generally advanced from the circumference towards the centre, sometimes this was not the case. The large fungus, which was seated on the thigh of Walsh, (Case II.) and which was at first without any crust, in the course of a few days became covered by two scabs, one on the upper and another on the lower part, and these separated by an interval which corresponded to the centre, and which healed first. In some cases, the semidiameter of a fungus will heal entirely, before the process of cicatrisation has commenced in the other half.

The number of fungi which are capable of existing on the same individual at the same time, is subject to great variety. I have seen cases when there was only one, and it is remarkable that on these occasions the fungus was, I believe, always extremely sensible. Sometimes I have seen as many as fifty on one patient, and I think it may be said that their magnitude is in general inversely as their number. Whether the fungi be great or small, few or numerous, they are, if not constantly, at least very uniformly of a circular figure. Sometimes two coalesce, and form the figure 8.

What the progress or termination of the disease would ultimately be, if left to itself, I cannot pretend to say, farther than to testify that it would be extremely tedious. Whether it would wear itself out or not, I cannot decide. On several occasions I have made efforts to watch its progress, uninfluenced by treatment, but its course has been uniformly so slow that the patient has become discontented, and I have been obliged to enter on its cure and removal before I could arrive at any satisfactory result. On other occasions, patients, who had been treated for this disease, have returned with the same affection; but whether this arose from any tendency which the disease has to relapse, or to the patient not having remained a sufficient length of time under treatment, I cannot determine, although I believe the latter to be the fact. I have not remarked that the disease becomes more intractable by long standing, or that it is more difficult to remove when it reappears after having been imperfectly treated.

In the whole catalogue of maladies which are capable of being cured or relieved by mercury, I do not know any which exhibits the value of this mineral more remarkably than the disease in question. I believe it matters nought whether this valuable agent be employed internally or externally. If internally, whether it be used as an oxide or salt: if externally, whether it be employed in the form of an unguent, vapour, or fluid; for the disease immediately shrinks as soon as the slightest mercurial action in the system is manifested. I generally retain the patients under care five or six weeks, and use the remedy to such an extent as to cause its gentle but marked influence on the system; and the form in which I employ it is varied according to the peculiar circumstances or convenience of each case.

Although mercury in some form or other, so as to affect the system gently, is the remedy which I now uniformly employ, and with which I always succeed, it is proper to remark that local applications to the fungi, which cannot be supposed to act on the general system, appear to have the power not only of controlling their growth, but also of causing their removal. The operation or influence of these topical remedies is however uniformly slow, frequently uncertain, and often causes the fungi to become extremely painful; and, although they may clear the surface of all the fungi which exist at the time of their application, the impression on my mind is that they do not destroy the disposition to the formation of new ones. Of the local means which I have found most serviceable, I may mention solutions of the sulphate of copper, of the nitrate of silver, and ointments of the subacetate of copper, of tar, and, above all, those unguents into which mercury enters as an ingredient. (*Ibid.*)

